# Developing a Deeper Understanding of Autism: Connecting Knowledge through Literature Mining

**DOI:** 10.1155/2011/307152

**Published:** 2011-06-07

**Authors:** Marta Macedoni-Lukšič, Ingrid Petrič, Bojan Cestnik, Tanja Urbančič

**Affiliations:** ^1^Department of Child Neurology, University Pediatric Hospital Ljubljana, Bohoričeva 20, 1000 Ljubljana, Slovenia; ^2^Centre for Systems and Information Technologies, University of Nova Gorica, Vipavska 13, 5000 Nova Gorica, Slovenia; ^3^Department of Knowledge Technologies, Jozef Stefan Institute, Jamova 39, 1000 Ljubljana, Slovenia

## Abstract

In the field of autism, an enormous increase in available information makes it very difficult to connect fragments of knowledge into a more coherent picture. We present a literature mining method, RaJoLink, to search for matched themes in unrelated literature that may contribute to a better understanding of complex pathological conditions, such as autism. 214 full text articles on autism, published in PubMed, served as a source of data. Using ontology construction, we identified the main concepts of what is already known about autism. Then, the RaJoLink method, based on Swanson's ABC model, was used to reveal potentially interesting, but not yet investigated, connections between different concepts in research. Among the more interesting concepts identified with RaJoLink in our study were calcineurin and NF-kappaB. Both terms can be linked to neuro-immune abnormalities in the brain of patients with autism. Further research is needed to provide stronger evidence about calcineurin and NF-kappaB involvement in autism. However, the analysis presented confirms that this method could support experts on their way towards discovering hidden relationships and towards a better understanding of the disorder.

## 1. Introduction

Autism spectrum disorders (ASDs) are currently one of the leading causes of developmental disability with approximately 1% children affected [1]. Etiologically, many different factors are involved. Among known genetic conditions that are associated with ASD in higher percentage compared with general population are fragile X syndrome (FXS), tuberous sclerosis, fragile premutation, phenylketonuria, 15q11-13 duplication, 16p11.2 duplication, and mutations in NGLN3, 4, phosphatase and tensin homolog (PTEN), and SHANK3, to name some of them. Better knowledge about the neurobiological basis of these disorders has led to many commonalities across them regarding underpinnings mechanisms and, most importantly, to potential targeted treatments [2–4]. 

One of recently most intensively studied area of gene-environmental interaction possibly involved in development of ASD is suspected immunological factors and processes. These factors include prenatal, genetic, and postnatal findings, as well as the discovery of a dysfunctional chronic proinflammatory state in brain tissue and cerebrospinal fluid in subsets of autistic patients [5]. Some genes, such as the tyrosine kinase receptor 7q31 metastasis receptor site (MET) gene, an immune-related gene affecting tyrosine kinase that can be involved in innate immune dysfunction, can double the risk of autism [6]. Other immune abnormalities possibly linked to autism are familial autoimmunity, maternal transfer of autoantibodies from the mother to child during pregnancy, production of antibodies against brain tissue in autistic patients, lower levels of normal immunoglobulins, and elevation of some cytokines [5]. Besides immune dysfunctions there are other epigenetic mechanisms potentially linked to autism such as increased level of oxidative stress, mitochondrial dysfunction, and excitotoxicity [2, 3]. 

Despite these very exciting new discoveries in the field of ASD, there are still a number of unique challenges including the heterogeneity of the disorder, the large number of symptoms that may be selected as targets for the therapy, and varying degrees of associated symptoms. Besides, ASD has to date been studied in several subfields and at several different levels, all using different procedures for examination: behavioural psychology, genetics, biochemistry, brain anatomy, physiology, and so forth. The question of how to connect partial results of individual sciences into a complete picture still remains very challenging. One method to manage increasing amounts of specialised knowledge and to support the process of its integration into a bigger and more coherent picture has been presented from knowledge technologies, and more specifically, from literature mining. This has become possible with the availability of huge online databases, such as PubMed, which covers over 20 million citations. A powerful idea for investigating yet to be explored relationships between biomedical concepts was proposed by Swanson [7]. If there is a relationship between A and B reported in the literature on A, and a relationship between B and C in literature on C, then the concept B, might reveal interesting connections across previously disjoint contexts A and C. Swanson found many relationships, unknown at the time, for example, connecting Raynaud's syndrome with fish oil, and migraine headaches with magnesium deficiency [7]. 

**Figure 1:**
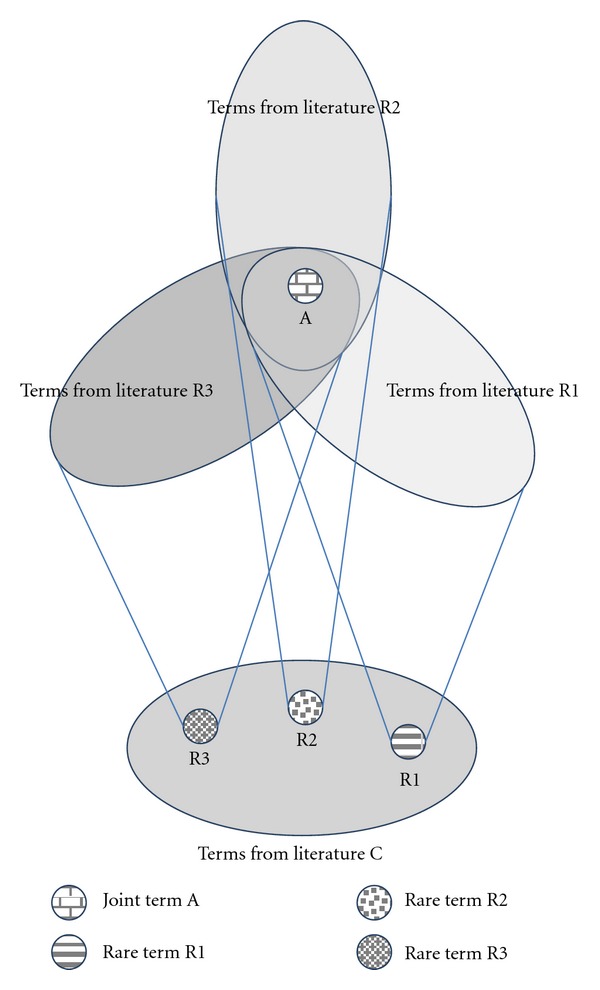
Principle behind RaJoLink.

The task of finding the intermediate concepts of B, when A and C are both already known, was defined as closed discovery [8, 9], as opposed to open discovery, where a search proceeds from C towards an unknown A. The latter is a crucial part of the scientific discovery process when generating new hypotheses. Therefore, our aim was to upgrade the hypothesis generation approach through a more systematic method in the open discovery stage. The basic idea was to use rare terms from the literature on the investigated phenomenon C as a guide toward new discoveries. This idea was first presented in Petrič et al. [10], as the RaJoLink method. This method has already been tested in the domain of autism [11, 12]. The main novelty regarding Swanson's method is the choice of candidates for A, which is based on rare terms identified in the literature on C. If the literature surrounding these rare terms has an interesting term in common, this joint term is declared as a candidate for A (see Figure 1). The underlying assumption is that while the majority of the literature in any given field describes matters related to common understanding of that field, particular observations appearing rarely in the literature, may provide a promising direction towards novel discoveries. The method is called RaJoLink, which is derived from its three basic steps.

1. Step *Ra*: Identification of interesting *rare* terms in the literature about C.
2. Step *Jo*: Search for a joint term, A, within the literature on the rare terms.
3. Step *Link*: Search for linking terms, B. For each B there should be a pair of articles, one from the literature on A and one from the literature about C, both mentioning B.

All three steps are implemented in a user-friendly computer program. In all steps, expert involvement is crucial for the selection of terms obtained from the automatically generated sets of candidate terms. This ensures that results are in line with the expert's interest and capture his or her expertise in guiding the whole process. Further technical details on each of the steps have been described previously [12].

The aim of this paper is to present and to analyse, in detail, findings obtained when applying the RaJoLink method in the field of autism. Our hypothesis is that using the RaJoLink method we can identify relationships between biomedical concepts in disconnected sets of articles which might lead to a better understanding of the possible causes of autism.

## 2. Methods and Materials

Publications about autism in the PubMed database served as a source of data. 10,821 publications were identified to August 21, 2006 as containing autis*, the expression root for autism. There were 354 full text articles published in the PubMed Central database. Due to a noticeable shift in the research focus within these investigations, we further restricted the inclusion to those published in the last ten years. This resulted in a final set of 214 publications.

Our examination of autism phenomena began with the identification of its main concepts and a review of what is already known about autism. We identified such information by ontology construction, which we found to be a very fast and effective way of describing the contents of large datasets. We used OntoGen [13], the interactive tool for semiautomatic ontology construction. OntoGen is based on machine learning and text mining techniques that automatically extract topics covered within the input documents. Thus, they support the user of the system to organise those documents into a topic ontology. Given that a biological basis to autism is nowadays broadly accepted, we began our autism research by focusing on very basic biochemical and neurological mechanisms. 

Using the RaJoLink system, approximately 2000 terms were automatically detected in the first step, Ra. Each of the terms appeared in only one of the publications from our set of 214. This long list of terms can be time consuming and confusing for further analysis. The RaJoLink ability to filter by MeSH (medical subject headings) classification (by choosing one or more top- or second-level MeSH categories) turned out to be very beneficial. In our experiments, we considered only the D12 second-level category from the 2008 MeSH tree structure, that is, amino acids, peptides, and proteins because, as mentioned, our research approach has been driven by biochemical mechanisms. Thus, we choose meaningful rare terms belonging to amino acids, peptides, and proteins, which in our experimental case included the terms lactoylglutathione, synaptophysin, and calcium channels. 

In the next step, Jo, we investigated whether the chosen terms, though rare in autism literature, pointed towards any interesting connections with autism in an indirect, yet logical way. We collated the PubMed abstracts of articles about lactoylglutathione, synaptophysin, and calcium channels into three separate text files and searched for terms that the three groups had in common. In this way, we singled out joint terms in the literature on the three rare terms. From several joint terms that were found automatically, calcineurin, a protein phosphatase that is widely present in mammalian brains, was chosen for further investigation.

## 3. Results

### 3.1. Autism and Calcineurin Relationship

We began the final step of our method by retrieving the abstracts of articles on autism as well as articles on calcineurin from the PubMed database. In this step, we found several pairs of PubMed articles that, when put together, could connect the two categories, as shown in Table 1.

It should be mentioned that all of the identified pairs of related publications, displayed in Table 1, proved to be very useful in guiding the discussion towards new ideas for further investigation.

Finally, we looked more closely at the significance of the fragile X protein loss in autism [14], due to the fact that fragile X syndrome has been found as one of the most recognisable causes for the disorder [15]. This evaluation helped us significantly in narrowing down our hypothesis. It encouraged us to further select data on autism and its relation to the fragile X. 

We found 41 full text articles in PubMed Central, which served as our input file of data on autism and fragile X. As in the case of our literature mining on pure autism articles, we used this new set of documents from PubMed in the open discovery stage for identification of rare terms. In a process similar to the one described previously, we found three interesting rare terms: BDNF (brain-derived neurotrophic factor), bicuculline, and c-Fos. In the next step, we found several promising joint terms in the intersection of the respective three sets of literature. One of these was the term NF-kappaB.

### 3.2. The Relationship between Autism and NF-kappaB

To test our hypothesis on the connection between autism and NF-kappaB we analysed the combined set of abstracts of 9,365 publications on autism and 30,893 publications on NF-kappaB. We found Bcl-2, cytokines, MCP-1, oxidative stress, and other meaningful terms linking the literature on autism with the literature on NF-kappaB (see Table 2).

## 4. Discussion

Using the literature mining method, RaJoLink, we aimed to uncover hidden connections that may provide additional clues about autism. In the first part of the study, calcineurin was identified as a possible important factor in the development of the disorder. The Ca^2+^/calmodulin-dependent phosphatase calcineurin has been shown to be involved in numerous diverse functions, both at the cellular and organism level. Its name is based on its calcium-binding properties as well as its abundant expression in the nervous system [16]. Several studies have supported the crucial role of calcineurin in modifying animal behaviours, predominantly by regulating cellular responses for timing and concentration of calcium in cells [17]. For example, it was shown that a reduction in function of calcineurin in mice resulted in defects in working/episodic like memory, hyperactive movement, social withdrawal, and defects in latent and prepulse inhibition [18]. 

In Table 1, literature on autism and calcineurin converge on the term synaptic plasticity (SP). This is a process that refers specifically to the activity-dependent modification of the efficacy in synaptic transmission at pre-existing synapses. For over a century, SP has been proposed as playing a central role in the capacity of the brain to incorporate transient experiences into persistent memory traces. Synaptic plasticity is also thought to play a key role in the early development of neural circuitry. Evidence is accumulating that impairment in SP mechanisms may contribute to several prominent neurodevelopmental disorders and among them is autism [19]. While so-called short-term plasticity is thought to play an important role in short-term adaptations to sensory imputes, transient changes in behavioural states, and short-lasting forms of memory, the “long-term” synaptic plasticity, in the form of long-term potentiation (LTP) and long-term depression (LTD), is thought to be, at least in part, the mechanism behind how an experience may modify a behaviour. Fragile X syndrome (FXS), the most common single gene cause of autism [4], linked to synaptic plasticity on the autism side of Table 1. FXS is caused by loss of function of fragile X mental retardation 1 gene (FMR1) resulted in absence of associated protein—FMRP, which is a RNA binding protein regulating translation of many target mRNAs. Most of the targets mRNAs studied so far have been linked to the regulation of synaptic function [20, 21], therefore we focused more closely on the correlation between calcineurin and FXS. 

Calcineurin is thought to be involved in N-methyl-D-aspartate receptors (NMDAR-) dependent LTD. This has mostly been studied in hippocampal cells, but was also found in neocortical synapses [22]. FXS, on the other hand has been linked to metabotropic glutamate receptor-(mGluR- ) dependent LTD, particularly in the hippocampus and cerebellum [14, 23]. This potential link between an mGluR-triggered form of SP and FXS has focused attention on mGluR antagonists as possible therapeutic agents for this and other developmental disorders [4, 24]. The fact that two different mechanisms of LTD involved in FXS and calcineurin action might be explained by an additive effect that both mechanisms have in children with FXS and autism compared to those with FXS without autism.

At present, there is no direct evidence reported on the role calcineurin plays in autism. Recently, however, evidence on the significant associations of a calcineurin isoform located at the chromosome 8p21.3 region and the subgroup of schizophrenia patients with deficits of sustained attention and executive functioning [25]. 

The second most important term found in our study was NF-kappaB. This transcription factor was first discovered by Sen and Baltimore [26] through its interaction with the immunoglobulin kappa light enhancer sequences. They referred to the binding site for this nuclear factor as the B site and therefore called the factor NF-*κ*B (nuclear factor-kappa B). 

Our results showed two points of convergences in the literature on both calcineurin and NF-*κ*B with autism. The first is Bcl-2, antiapoptotic protein, that works mainly at the level of mitochondria [27]. Fatemi and colleagues [28] reported a 34% to 51% reduction in Bcl-2 levels in the cerebellum of autistic patients compared with controls. Their experiments showed that deregulation of Bcl-2 may result in some of the structural brain and behavioural abnormalities in patients with autism. On one hand, it has been shown that activation of NF-kappaB in neurons can promote their survival by inducing the expression of genes encoding Bcl-2 [29] and on the other, that calcineurin occurred as a complex with Bcl-2 in various regions of rat and mouse brains, in particular during times of cellular stress and damage [30]. 

More specifically the protective role of Bcl-2 in metabolic oxidative stress-induced cell death was showed by Lee and colleagues [31]. The problem of oxidative stress in autism has been extensively studied [32]. Also, over the last decade there has been increasing evidence about the association between autism and mitochondrial dysfunction [33, 34]. As Bcl-2 works as an antiapoptotic agent mainly at the level of mitochondria, we may propose that its downregulation is one of the mechanisms related to increase of oxidative stress and mitochondrial dysfunction in autism. Additionally, it has been shown that FMRP expression is, among other functions, an essential part of cellular survival mechanisms, at least partly through modulation of Bcl-2 signal pathways [35].

The second point of convergence was a possible immunomodulatory role of both substances, calcineurin, and NF-kappaB. Neuroimmune abnormalities in the brain of patients with autism have recently become objects of growing scientific interest [36–38]. It has been proposed that abnormalities in innate as well as in adaptive immune responses play a potentially important role in the development of autism [36, 37]. NF-kappa B has been shown to have a crucial and multifaceted role in innate immune responses [39]. Further, it has been specified that, on activation, NF-kappaB regulates the expression of almost 400 different genes, which also include the inflammatory cytokines, such as TNF, IL-1, IL-6, IL-8, and chemokines [40]. NF-kappaB is also involved in macrophage chemoattractant protein (MCP-1) gene expression as an intracellular signalling mechanism [41]. Vargas and colleagues [37] indicated that MCP-1 is one of the most prevalent cytokines in brain tissue from patients with autism.

A better understanding of neuroinflammation in the pathogenesis of autism may have important clinical and therapeutic implications. So far, immune modulation and therapy, which have been applied to individuals with autism, are mainly limited to a number of case reports and no consensus treatment guidelines for immune therapies have been developed to physicians to use. Among the current studies of immune-targeted therapies, the collective data on steroid effects on autism is the largest, mainly in the subgroup of patients with EEG abnormalities that go together with autistic regression [5]. 

Currently, the US National Institute of Health Clinical Center is recruiting participants to test the effectiveness of minocycline in treating regressive autism through a blockade of NF-kappaB nuclear translocation. The anti-inflammatory antibiotic, minocycline, is a potent inhibitor of microglial activation, which reduces inflammation by blocking the nuclear translocation of the proinflammatory transcription factor, NF-kappaB. Also, there's an evidence about minocycline beneficial effects in patients with FXS [42].

Calcineurin is also a common target of the immunosuppressant drugs cyclophilin-cyclosporin A and FKBP-FK506 complexes [43]. Its inhibition has also been tested to achieve successful immunosuppression in patients after organ transplantation and in treating several other medical problems [44]. 

It is thought that autism results from an interaction between genetic and environmental factors with immunological disorders as one of the potential mechanisms linking the two [45, 46]. Our results, with an emphasis on calcineurin and NF-kappaB, add further reasons for the need of more research into possible neuroimmune factors in the development of autism and as potential novel targets for treatments.

## 5. Conclusions

In this study, it has been shown how the literature mining method can support medical experts on their way to discovering hidden relationships in data and a deeper understanding of complex disorders such as autism. The method has been proven as a very important tool, especially in the time of a vast amount of information at differing levels of knowledge. Also, through the interaction of different professionals the entire process of knowledge discovery can benefit, by faster speed and guidance towards more meaningful solutions. An important feature of the two agents that emerged as potential links to autism in our study, calcineurin and NF-kappa, need to be further investigated to gain stronger evidence on their involvement in the development of the disorder.

## Figures and Tables

**Table 1:** Potential hypotheses for the relationship between autism and calcineurin.

Autism literature	Calcineurin literature
Fatemi [28] reported a reduction of *Bcl-2* (a regulatory protein for control of programmed brain cell death) levels in the cerebellum of patients with autism.	Erin et al. [30] observed that calcineurin occurred as a complex with *Bcl-2* in various regions of rat and mouse brains.

Roesler et al. [47] reported on a translocation in the GRPR gene, the mammalian bombesin-like gastrin-releasing peptide, being associated with autism.	Corral et al. [48] showed that bombesin promotes the activation of the nuclear factor of activated T cells (NFAT) through a Ca^(2+)^/calcineurin-linked pathway.

Huber et al. [14] showed evidence of an important functional role of fragile X protein, an identified cause of autism, in regulating activity-dependent synaptic plasticity in the brain.	Winder and Sweatt [49] described the critical role of protein phosphatase 1, protein phosphatase 2A, and calcineurin in the activity-dependent alterations of synaptic plasticity.

Belmonte et al. [45] reviewed neuropathological studies of the cerebral cortex in autism indicating abnormal synaptic and columnar structure and neuronal migration defects.	Chen et al. [50] reported about the decrease in protein ubiquitination in synaptosomes and in nonneuronal cells that may play a role in the regulation of synaptic function by a calcineurin antagonist FK506.

Vorstman et al. [51] stated that autism spectrum disorders and subthreshold autistic symptoms are common in children with 22q11.2 deletion syndrome.	Sivagnanasundaram et al. [52] examined the differential expression of gene mapping to human chromosome 22q11.2 in 22q11.2 deletion syndrome and found the decreased expression of calmodulin 1 encoding a calcium-dependent protein involved in the calmodulin-calcineurin regulated pathway, which is implicated in learning and memory.

Mouridsen et al. [53] observed two autoimmune conditions associated with infantile autism: ulcerative colitis in mothers and type 1 diabetes in fathers.	Winter and Schatz [54] listed immunosuppression by calcineurin inhibitors as one of the promising strategies for intervention in autoimmune type 1 diabetes mellitus.

Román [55] proposed that morphological brain changes in autism may be produced by maternal hypothyroxinemia resulting in low triiodothyronine in the foetal brain during pregnancy.	Sinha et al. [56] found that calcineurin was compromised in young progeny when they investigated the maternal hypothyroxinemia effect during pregnancy on the brains of young progeny.

Omura [57] published the results of measurements of asbestos accumulation where relatively high levels of asbestos were found in autism.	Li et al. [58] investigated the role of reactive oxygen species, by asbestos, in activation of nuclear factor of activated T cells (NFAT). They found that pretreatment of cells with cyclosporin A, a pharmacological inhibitor of calcineurin, blocked asbestos-induced NFAT activation.

Thornton [59] argued that artificially generated electromagnetic radiation may play an important role in the mirror neuron dysfunction associated with autism.	Manikonda et al. [60] indicated that exposure to extremely low-frequency electromagnetic fields caused increased activity of calcineurin in the hippocampal region of rats.

Barnard et al. [61] revealed that adults with autism showed impaired performance on the tests of working memory.	Runyan et al. [62] illustrated how the inhibition of calcium activated phosphatase calcineurin causes impaired working memory.

**Table 2:** Potential hypotheses for the relationship between autism and NF-kappaB.

Autism literature	NF-kappaB literature
Araghi-Niknam and Fatemi [63] showed a reduction of *Bcl-2*, an important marker of apoptosis, in the frontal, parietal, and cerebellar cortices of autistic individuals.	Mattson [29] reported that activation of NF-kappaB in neurons can promote their survival by inducing the expression of genes encoding antiapoptotic proteins such as *Bcl-2* and the antioxidant enzyme Mn-superoxide dismutase.

Vargas et al. [37] reported altered *cytokine* expression profiles in brain tissue and cerebrospinal fluid of patients with autism.	Ahn and Aggarwal [40] reported that, on activation, NF-kappaB regulates the expression of almost 400 different genes, which include enzymes, *cytokines* (such as TNF, IL-1, IL-6, IL-8, and chemokines), adhesion molecules, cell cycle regulatory molecules, viral proteins, and angiogenic factors.

Vargas et al. [37] also indicated that macrophage chemoattractant protein MCP-1 and tumor growth factor-beta1 were the most prevalent cytokines in the brain tissue of patients with autism.	Thibeault et al. [41] showed that the MCP-1 gene is expressed within particular populations of cells in response to inflammatory molecules that employ NF-kappaB as an intracellular signaling mechanism.

Ming et al. [46] reported on increased urinary excretion of an oxidative stress biomarker—8-iso-PGF2alpha in autism.	Zou and Crews [64] reported an increase in NF-kappaB DNA binding following oxidative stress neurotoxicity.

Yoo et al. [65] observed statistically significant associations between polymorphisms of PTGS2, the gene encoding cyclooxygenase-2, and autism spectrum disorders.	Lee et al. [66] elucidated the role of spinal NF-kappaB in the cyclooxygenase-2 upregulation and pain hypersensitivity following peripheral inflammation.

Ma et al. [67] performed a genome-wide linkage analysis on 26 extended autism families and found significant linkage to chromosome 12q14.	Weersma et al. [68] mentioned chromosome 12q14 as a region of IRAK-M gene, which is an NF-kappaB-mediated negative regulator of the toll-like receptor/IL-1R pathways.

Steele et al. [69] demonstrated spatial memory deficits in high-functioning individuals with autism, particularly as tasks required heavier demands on working memory.	Denis-Donini et al. [70] highlighted the function of NF-kappaB in hippocampal neurogenesis and in short-term spatial memory.

Jyonouchi et al. [71] revealed intrinsic defects of innate immune responses in children with autism spectrum disorders and gastrointestinal symptoms.	Thomas et al. [39] confirmed that NF-kappaB has a crucial and multifaceted role in innate immune responses.

Grigorenko et al. [72] identified macrophage migration inhibitor factor, which is an upstream regulator of innate immunity, as a possible susceptibility gene for autism spectrum disorders.	Gore et al. [73] showed that macrophage migration inhibitor factor regulates subsequent adaptive immune responses by initiating a signalling cascade that activates NF-kappaB.

Johnson and Malow [74] highlighted frequent sleep problems among children with autism, such as obstructive sleep apnoea.	Yamauchi et al. [75] identified significantly greater activation of NF-kappaB, which occurred in obstructive sleep apnoea.

## Abbreviations

ICD-10: International Classification of Diseases
DSM-IV: Diagnostic and Statistical Manual of Mental Disorders
PRM: Pervasive developmental disorders
ASD: Autism spectrum disorders
MeSH: Medical subject headings.
